# Clear Plaque Mutants of Lactococcal Phage TP901-1

**DOI:** 10.1371/journal.pone.0155233

**Published:** 2016-06-03

**Authors:** Witold Kot, Mogens Kilstrup, Finn K. Vogensen, Karin Hammer

**Affiliations:** 1 Department of Environmental Science, Aarhus University, Frederiksborgvej 399, DK-4000, Roskilde, Denmark; 2 Department of Systems Biology, The Technical University of Denmark, Matematiktorvet Building 301, DK-2800, Lyngby, Denmark; 3 Department of Food Science, University of Copenhagen, Rolighedsvej 26, DK-1958, Frederiksberg C, Denmark; Niels Bohr Institute, DENMARK

## Abstract

We report a method for obtaining turbid plaques of the lactococcal bacteriophage TP901-1 and its derivative TP901-BC1034. We have further used the method to isolate clear plaque mutants of this phage. Analysis of 8 such mutants that were unable to lysogenize the host included whole genome resequencing. Four of the mutants had different mutations in structural genes with no relation to the genetic switch. However all 8 mutants had a mutation in the *cI* repressor gene region. Three of these were located in the promoter and Shine-Dalgarno sequences and five in the N-terminal part of the encoded CI protein involved in the DNA binding. The conclusion is that *cI* is the only gene involved in clear plaque formation i.e. the CI protein is the determining factor for the lysogenic pathway and its maintenance in the lactococcal phage TP901-1.

## Introduction

The birth of phage genetics was based on the visual inspection of the morphology of plaques. Isolations of clear plaques mutants have been the classic route for obtaining mutations in the major regulators responsible for immunity. The model for temperate phages, bacteriophage lambda, forms turbid plaques on its *Escherichia coli* host, and this phenotype was used for isolation of clear plaque mutants (CI) more than fifty years ago[1]. In accordance with the nomenclature in lambda, phage genes in other phages coding for repressors of the lytic cycle are usually named *cI*. The isolation and analysis of such clear plaque mutants has also been used to identify additional genes involved in the maintenance of the lysogenic state or in the decision process leading to lysogenisation. In lambda, mutations in three different genes *cI*, *cII* and *cIII*[1] were found to result in clear plaques. The CI protein is necessary for establishment and maintenance of lysogenic state, the activator CII is necessary for efficient establishment of lysogeny, and CIII is needed for increasing the stability of the otherwise unstable CII protein[2]. The lactococcal temperate phage TP901-1 has an interesting and simple switch mechanism consisting of a repressor CI and a small protein, Mor, which apparently is counteracting CI repression of the lytic promoter P_L_[3]. CI and Mor are expressed from two divergent promoters. When a 1kb DNA fragment containing the entire switch region is inserted into a plasmid, transformation of *Lactococcus lactis* will lead to two alternative stable phenotypes with the lytic promoter being either open or closed, thus mimicking the choice after phage infection.

In the study of the genetic switch of TP901-1 we wanted to isolate and analyze clear plaque mutants to investigate if genes other than CI could be involved in the decision process. This procedure had never been used before because the wild type TP901-1 forms clear plaques on its host *Lactococcus lactis* subsp. *cremoris* 3107 using standard plating procedures. We expected that the reason for the lack of turbidity was caused by the inability of the immune lysogens to grow in the plaques due to the low pH produced by the lactic acid bacterial lawn. Hence a reduction in the acidity of the plates during growth of the host layer might allow for further growth of the lysogens allowing formation of a turbid plaque. Adding additional MOPS buffer to the plates and reducing the glucose concentration resulted in slightly turbid plaques of the parent phage and allowed the selection of clear plaques. Isolation and characterization of the clear plaque mutants resulted in mutations only in the *cI* gene and thus supports the model for the TP901-1 phage that the CI repressor is the determining factor in lysogeny and no other gene product is necessary for its synthesis in TP901-1. The CI protein, transcribed from the P_R_ promoter in TP901-1, has been purified and shown to form a hexamer in solution. The DNA binding domain is located in the N-terminal part of the 180 amino acid long CI monomer, while the C-terminal end is responsible for the oligomerization [4]. The three dimensional structure of the N-terminus of CI has been determined [5], Mor (Modulator of Repression) is a small protein of 72 amino acids, it is transcribed from P_L_ (the lytic promoter in TP901-1), which is located in the opposite direction of P_R_. Using transcriptional fusions it has been shown that a surplus of Mor counteracts repression by CI at P_L_, but also increases repression by CI at P_R_ [3]. CI-Mor protein-protein interactions have been proposed to be responsible for this behaviour [5,6].

## Methods

### Growth medium

M17 with 1% glucose (GM17) was used for growth of the lactococci [7], except when clear plaques were isolated, in that case M17/7 was used. M17/7 corresponds to M17 supplemented with 200mM MOPS instead of beta-glycerophosphate and adjusted to pH 7.2 with NaOH, glucose was added to 0.5%[8]. SAL is a defined SA medium[9] where the Sodium Acetate has been substituted with 2μg/ml lipoic acid. *E*. *coli* strains were grown in LB medium [9] with selection for erythomycin at 150 μg/ml.

### Bacterial stains, plasmids and phages

The bacterial strains and plasmids used are found in Table 1.

**Table 1:** Plasmids and strains used in this study.

Plasmid or strain	Description	Reference or Source
**pLB86**	Promoter probe vector containing *attP* (TP901-1) and used for integration in *Lactococcus lactis*	Brøndsted and Hammer [10]
**pMK1228**	pLB86 containing *cI* P_R_ O_R_ O_L_ P_L_	This work
**pMK1223**	pLB86 containing *cI* with mutation C3, P_R_ O_R_ O_L_ P_L_	This work
**pMK1224**	pLB86 containing *cI* with mutation C8, P_R_ O_R_ O_L_ P_L_	This work
**pMK1231**	pLB86 containing *cI* with mutation C18, P_R_ O_R_ O_L_ P_L_	This work
**LB504**	*Lactococcus lactis* subsp *cremoris* MG1363 / pLB95 containing the *int* (TP901-1) used for integration of pLB86 derivatives	Brøndsted and Hammer [10]
**MK755**	LB 504 *attB*::pMK1228	This work
**MK750**	LB 504 *attB*::pMK1223	This work
**MK751**	LB 504 *attB*::pMK1224	This work
**MK763**	LB 504 *attB*::pMK1231	This work
***Lactococcus lactis* subsp *cremoris* 3107**	Host for TP901-1 and TP901-BC1034	Braun et al. [11]
***Escherichia coli* MC1061**	Used as host for plasmids during cloning	Casadaban and Cohen [12]

Lactococcal bacteriophage TP901-1[13] and TP901-BC1034[14] were used. The TP901-BC1034 is a TP901-1 derivative, which encodes erythromycin resistance and was used to aid measurement of lysogenization.

### Mutagenesis and isolation of clear plaque mutants

The TP901-BC1034 phage stock was diluted in 0.9% NaCl and mutagenized by UV light resulting in a 50 fold reduction in titer. The mutagenized stock was wrapped in foil and kept in the dark until use the next day.

The host *L*. *lactis* subsp. *cremoris* 3107 was grown exponentially until OD_600_ of 1.0 in M17/7, then it was diluted in 0.9% NaCl and mutagenized by UV for 12 seconds, this affected viability with a factor 2 or less. The UV treatment was performed in order to induce the SOS system in the host and hence increase the mutagenic effect of the damaged phage DNA. The bacteria were used immediately for plaquing using the mutagenized phage stock. The mixture was incubated for 10 min at 30°C and then plated in M17 top agar with 0.5% agar and 5mM CaCl_2_ on M17/7 plates containing 1.1% agar and 5 mM CaCl_2_; they were incubated anaerobically for approx. 24h at 30°C, aerobic incubation gave slightly turbid plaques.

### Lysate

Single plaque lysate was made using a fresh plaque to which 0,1 ml O/N culture of *L*. *lactis* 3107 and 5 mM CaCl_2_ was added. After 5–10 min at room temp, 10 ml GM17 with 5 mM CaCl_2_ was added and the mixture was incubated at 30°C until lysis occurred. Lysates were filtered using Q-Max Syringe filter with a pore size of 0.45 μm.

### DNA preparation

Phage DNA from clear plaque mutants and from the wild type phage TP901-BC1034 that was used for mutagenesis was isolated from CsCl purified phage preparations by phenol-chloroform extraction as described previously for phage lambda by Sambrook and Russell[15].

### Sequencing and sequence analysis

DNA sequencing libraries were prepared using the Nextera® XT DNA kit (Illumina, San Diego, USA) according to the manufacturer’s protocol. Individually tagged libraries were sequenced as a part of a flowcell as 2 × 250 bases paired-end reads using the Illumina MiSeq platform (Illumina, San Diego, USA). Reads were trimmed, analyzed and assembled using CLC Genomic Workbench 7.5 (CLCbio, Aarhus, Denmark). Briefly, reads were trimmed (0.05 quality limit) and mapped to reference (length fraction = 0.5, similarity fraction = 0.8, reference accession- AF304433). Afterwards reads mapping were searched for mutations using the probabilistic variant detection tool followed by filtering variants against control reads derived from the TP901-BC1034 phage.

### Lysogenization

*Lactococcus lactis* subsp. *cremoris* 3107 was grown at 30°C in GM17+ 0.5% glucose. When the growth was exponential at OD_600_ 0.8–1.0, CaCl_2_ was added to 10 mM and the culture was diluted ten fold in prewarmed medium. TP901-BC1034 phages carrying erythromycin resistance were added at a multiplicity of 0.2 and incubated for 35 min. Topagar containing 20 mM sodium citrate was added and the mixture plated on GM17 plates containing 10 mM citrate and 2 μg erythromycin selecting for erm resistant transductants. Using these conditions the wild type phage TP901-BC1034 gave 2 transductants per 10^3^ infected bacteria.

### Analysis of CI repression of the P_L_ promoter

Promoter fusions were created in the integration plasmid pLB86 [10] by PCR amplification of the region from base pair 2627 to 3351 in TP901-BC1034 containing *cI*, P_R_, O_R_, O_L_ and P_L_ from wild type and mutant phages. Purified phage chromosomal DNA and primers MK638 (5’-AAAAAAGCTTCTCGAGAAAGCTCTCTAAG-3’) and MK660 (5’-AAAACTGCAGTTTCTCCTTTCTTTCAGTTCACGTTTCAT-3’) were used, resulting in DNA fragments containing the *cI* gene expressed from its own P_R_ promoter, and the P_L_ promoter directed towards a PstI restriction site. Insertion of the PCR fragments into the pLB86 transcriptional fusion plasmid by use of the XhoI and PstI restriction sites followed by transformation of *E*. *coli* MC1061 resulted in plasmids pMK1228, pMK1223, pMK1224, and pMK1231 for the wild type, C3, C8, and C18 mutant phages, respectively. In all plasmids, the P_L_ promoter was inserted in front of the promoterless *lacLM* reporter. Following plasmid preparation and transformation of *L*. *lactis* strain LB504, the strains MK755 (LB504 *attB*::pMK12528 (wt), MK750 (LB504 *attB*::pMK1223 (C3), MK751 (LB504 *attB*::pMK1224 (C8), and MK763 (LB504 *attB*::pMK1231 (C18)) were created, in which the fusion plasmids had integrated into the *attB* site on the chromosome.

Fusion strains were cultured in triplicate (inoculated from individual bacterial colonies) at 37°C in SAL medium containing 1% glucose and erythromycin at 2 μg/ml. Dilutions were prepared to ensure exponential growth for at least 8 generation, and each culture was harvested after approximately 8, 11, and 14 generations. The resulting nine samples for each strain were harvested and assayed for β-galactosidase activity as described earlier[6] except that the specific activity was based upon OD_450_ instead of OD_600_.

## Results

### Reduced acidification of plates results in turbid plaques of TP901-BC1034

The standard plating procedure for obtaining TP901-1 plaques results in clear plaques, which has prevented the selection of CI mutants based upon plaque turbidity. Since a large amount of lactic acid is produced during growth of *L*. *lactis*, we tried to prolong the growth of the bacterial lawn by counteracting the lowering of the pH through a combination of higher buffer capacity and lower sugar availability in the medium. Addition of 200 mM MOPS pH 7.2 and lowering of the glucose concentration from 1% to 0.5% and anaerobic incubation had the required effect and we obtained large turbid plaques upon infection of *L*. *lactis* strain 3107 with the wild type bacteriophage TP901-1 as well as the TP901-BC1034 labelled with an Erm^R^ cassette. No clear plaques were identified by screening more than 1000 plaques from a normal phage stock.

### Mutagenization and screening for clear plaques with lowered lysogenization efficiency

After UV mutagenesis of a TP901-BC1034 phage stock with reduction of the phage titer to 2%, clear plaques were found at a frequency of 0.1%. The plaques were purified to single plaques 3 times for obtaining a stable clear plaque phenotype. Lysates prepared from 8 such mutants (C3, C6, C8, C9, C18, C23, C25, and C26) were used for lysogenization tests and phage genome sequencing.

Lysogenization was analysed by measuring the efficiency of transducing the Erm^R^ resistance cassette, harboured in TP901-BC1034, to the host *L*. *lactis* 3107. It was shown that all mutant phages were unable to lysogenize (less than 10^−6^ to 10^−8^ transductants per infected bacterium)

### Genome sequencing and identification of clear plaque mutations

DNA was extracted from the phage lysates and the genome sequences were determined. The genomic sequence of the parent TP901-BC1034 phage used in this experiment exhibited changes in comparison to the GenBank reference of TP901-1 (AF304433). One of them was the insertion of an Erm^R^ cassette in ORF56_TP901-1_, as reported earlier for TP901-BC1034[14]. The second rearrangement region was more complex and was localized in the region responsible for morphogenesis of the phage, precisely in region ranging from the *orf31* coding for large terminase subunit (*terL*) to *orf36* coding for major head protein (*mhp*). We reasoned that the second rearrangement was likely to be caused by homologous recombination with a prophage during the Erm^R^ labelling process or while maintaining the TP901-BC1034. Sequencing and analysis of the host genome, *L*. *lactis* subsp. *cremoris* 3107, indeed revealed that it has a prophage sequence that could recombine with TP901-BC1034 (data not shown). In order to see if the recombination between 3107 and TP901-1 had taken place we designed a PCR test, which verified that TP901-BC1034 has a fragment of a prophage from 3107. The rearrangement was regarded as irrelevant for the clear plaque morphology as it was present in both the parent phage TP901-BC1034 (turbid plaques) and all the clear plaque mutants. S1 Fig and S1 Table show the sequence differences between the mutants and the parent phage TP901-BC1034 sequence together with the region of recombination between TP901-1 and a 3107 prophage. Half of the mutant phages were single mutants (C3, C8, C18, and C26) while the other half had additional mutations in the late structural genes (S1 Table).

Due to the late expression of these genes the mutations were not likely to influence the regulatory switch determining the lysogenization of the phage. As the phage mutants were able to form fine plaques it was known that the mutations did not affect the viability of the phages either. C9 had amino acid changes in the C-terminal end of the integrase gene (Leu-Gly to Leu-Ser change) in addition to a mutation in the end of the *nps* structural gene coding for collar-whisker structure. Deletion of A in *nps* resulted in frame shift mutation from 433 amino acid. Collar whisker structure was shown before not to be an essential part of a TP901-1 virion structure[16]. A mutation in *int* might affect lysogenization of C9, however it has a secondary frameshift mutation located in the *cI* gene. This mutation is located just besides the position of a single frameshift mutation in C26 with a similar phenotype, therefore it is more likely that the mutation in *cI* is responsible for the observed phenotype of C9. C23 had 3 mutations in the *tal* gene encoding the tail-associated lysis protein[17] (positions in TP901-1 27069–27089), which resulted in Gln-Lys, Ser-Tyr and Gly-Asp substitutions.

**Fig 1:**
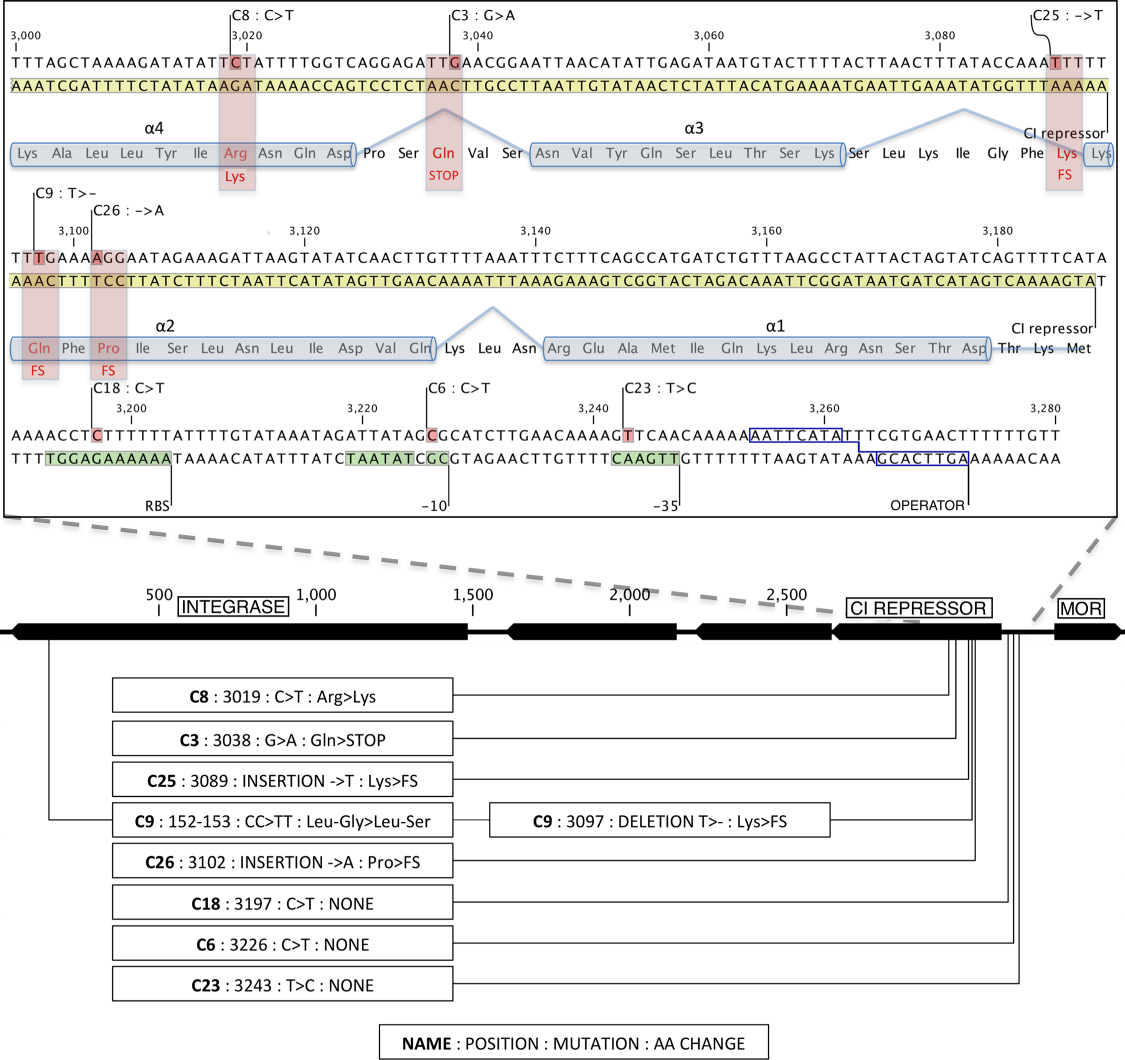
Localization of TP901-BC1034 clear plaque mutations. Expanded view of the region from position 0 to 3600 showing the clear plaque mutations. A physical map of orf1 (Integrase) to orf5 (Mor) is shown in the middle with a size indicator. Below this each mutation is identified in boxes according to its [designation: precise position: nucleotide change: change at the amino acid level (STOP = nonsense mutation; FS = frameshift mutation; NONE = no change in amino acid sequence)]. Above the physical map is a panel showing the genetic context of the CI mutations. Below the nucleotide sequence the amino acid sequence is indicated with boxing of amino acids that are believed to form alpha helical structures (alpha1 to alpha 4) in the wild type CI protein [5]. Extended promoter -10 and -35 elements and the ribosomal binding site (RBS) are highlighted in green. The O_R_ site is indicated in blue box (operator). The O_L_ site is located 63 bp further to the right of the O_R_ site overlapping the start of the O_L_ transcript (not shown).

No attempts were made to separate the mutations in the double and triple mutants because all eight mutant phages had mutations in the *cI* region and these are highly likely to result in clear plaque phenotypes. In the lower part of Fig 1, it can be seen that mutant C3 had acquired an early nonsense mutation in the *cI* gene, and that the C9, C25 and C26 mutants had early frame shift mutations. None of these mutations would be expected to result in functional CI proteins since the changes occurred either in the DNA binding motif (helix α2 and helix α3 in Fig 1) or just after.

The stop codon in C3 mutant is located only two amino acids after helix α3, the DNA recognition helix. The C23 mutant had a mutation that changed the -35 region of the P_R_ promoter from TTGAAC to TTGAGC. Since a G in position 5 of the -35 element is the least common nucleotide for RpoD promoters [18], the mutation is likely to lower the promoter activity. The C6 mutant changed the sequence immediately upstream of the -10 element in the P_R_ promoter from TGCGCTATAAT to TGCACTATAAT. Promoters with extended -10 regions (TaTGgTATAAT) are known to be much stronger than their non-extended counterparts [19], so the C6 mutation is consistent with a weaker P_R_ promoter. Along the same lines, the C18 mutant had acquired a mutation that changed its SD sequence AAAAGAGG to AAAAAAGG, fitting the consensus of the ribosomal binding site to a lesser degree. The C6, C18, and C23 mutants are therefore likely to express lower levels of CI compared to the wild type phage therefore being unable to lysogenize. No mutations were found in the binding sites for CI, O_R_, O_L_ and O_D_

The most unexpected mutant is C8 that has acquired a conservative mutation, changing the amino acid 57 from arginine to a lysine. It appeared strange that this mutation should render the CI protein unable to repress, as the altered amino acid is distant from the DNA binding region. However, the region where the C8 mutation is located has previously been proposed to be involved in binding to the small regulatory Mor protein, which has been suggested to function as an antirepressor for CI repression at the P_L_ promoter [3]. The proposed Mor binding region in CI is based upon homology to other CI-Mor partners and the non repressible phenotype in the presence of Mor of two 5 amino acid insertion mutations in CI [5,6]. It might be possible that a *cI* mutation could result in a clear plaque phenotype if it resulted in increased binding between CI and Mor without disrupting CI repression in the absence of Mor.

### The mutation in C8 does not inactivate P_L_ repression in the absence of the Mor protein

Because the C8 mutant was a clear plaque mutant, we knew that CI was not able to repress in the presence of Mor. To pursue the possibility that the C8 mutation was involved in Mor binding we had to show that the C8 mutation did not prevent CI repression of P_L_ in the absence of Mor. From the wild type phage, and the C3, C8, and C18 mutants we amplified the *cI* gene with its own promoter (P_R_) and the adjacent P_L_ promoter, but without the *mor* gene. After insertion into plasmid pLB86, with P_L_ upstream from the promoterless *lacLM* gene, and integration of the fusion plasmids into the *attP*_TP901-1_ locus on the chromosome of *L*. *lactis* LB504, the fragments were assayed for P_L_ promoter activity. The cloned piece of DNA contains O_R_ and O_L_, but not O_D_ (Table 1). According to Pedersen and Hammer [20], P_L_ is repressed almost the same on the plasmid containing O_R_ and O_L_, but without O_D_, as in the presence of all three sites. Assuming the P_L_ activity seen with the C3 mutant (with a stop codon early in the cI gene) represents fully unrepressed P_L_, the wild type fusion gives ~1000-fold repression (Table 2). Although the C8 mutation was slightly de-repressed (6-fold compared to the wildtype), it still retained 160-fold repression indicating that the mutant repressor is still functional in the absence of Mor. Interestingly the SD mutation in C18 retained 260-fold repression of P_L_ in the absence of Mor, despite the fact that the mutation result in a clear plaque phenotype. This may be explained by the presence of a wild type functional CI repressor combined with the autoregulation of the P_R_ promoter by CI. The reduced translation efficiency caused by the C18 mutation is outbalanced by increased expression from the P_R_ promoter. However in the phage it results in a clear plaque. This shows that the expression levels of CI and Mor in the wild type phage have to be finely tuned to produce the normal lysogenization frequency.

**Table 2:** Analysis of CI repression in P_L_-*lacLM* fusions. The *cI* region from wt TP901-1-BC1034 and mutants C3, C8, C18 were inserted in plasmid pLB86 and integrated into the *attB* locus of *L*. *lactis* subsp. *cremoris* LB504. Three biological replicates, each determined after exponential growth for approximately 10, 13 and 16 generations. Activity is given as 1000 x A_420_/(min x OD_450_).

Strain	cI mutation	β-galactosidase activity (+/- SD)*	Repression fold
**MK755**	None	0.3 (+/- 0.1)	1000
**MK750**	Nonsense (C3)	310 (+/- 70)	1
**MK751**	R57K (C8)	2.0 (+/- 0.5)	160
**MK763**	SD (C18)	1.2 (+/- 0.4)	260

## Discussion

Temperate bacteriophages have been found to have a significant impact on bacterial evolution[21]. The study of the genetic switches are therefore important for understanding of the transfer mechanisms. Such a study usually implies isolation of clear plaque mutants in the phage.

A clear plaque mutant in a temperate lactococcal phage has to our knowledge so far only been isolated in φLC3[22]. The mutant had lost its ability to lysogenize and later DNA sequencing[23] showed that it had acquired a stop codon in amino acid 197 in the repressor protein (total size 286 amino acids).

In order to get turbid plaques of the wild type TP901-1 on its host strain, we first had to design a modified growth medium. Afterwards we then succeeded in isolating clear plaque mutants of TP901-BC1034 after UV mutagenesis. We analysed 8 mutants further as reported in results.

They were all defective in lysogenization and they all had mutations in the *cI* gene. The phages were fully genome sequenced and four of them had additional mutations (S1 Table). Of these only the *int* mutation in C9 might affect lysogenization, all other mutations in C6, 23 and 25 were in late structural genes with no relevance for lysogenization.

All 8 *cI* mutations map in the first third of the *cI* gene region, three of them in the promoter and the Shine-Dalgarno sequence, respectively. Three of the remaining 5 mutations within the structural gene give rise to frame shift mutations before or in the DNA binding motive (C26, C9 and C25). One, C3, gives rise to a stop codon just after the DNA binding motive and the last mutation C8 is a conservative amino acid exchange from Arg to Lys in amino acid 57, located 9 amino acids after the DNA binding motive.

The localization of the mutations might indicate that the last part of CI is not necessary for DNA binding. This is in accordance with previous results, that 5 amino acid insertions in the CI protein from amino acid 78 and further downstream do not abolish repression of the lytic P_L_ promoter[6]. The purified CI protein has been shown to be a hexamer in solution. However when the last 43 amino acids in the C-terminal end are removed, it forms mainly a dimer, but the truncated CI protein is still able to repress the P_L_ promoter on a plasmid containing the switch region with the O_L_ and O_R_ sites [4]. Our results presented here may also indicate that multimerization to a hexameric form is not needed for lysogen formation but probably results in more stable lysogens.

No mutations were found in the three operator regions. The O_R_ site, located between the P_R_ and the P_L_ promoter, is a poor binding site for CI, while the O_L_ site located in the start of the transcript from P_L_ is a strong binding site, this is also the case for the O_D_ site located at the end of the Mor transcript. When only O_L_ is present in plasmid P_L_ is also repressed, however a O_L_ mutation alone does not abolish P_L_ repression if both O_R_ and O_D_ is present [20]. This is probably the reason why none of the phage mutants obtained had mutations in O_L_, since it would have required an additional mutation in O_D_ or O_R_.

The finding that only mutations in *cI* are responsible for the clear plaque phenotype strongly indicates that only CI is required for the choice of lysogenization and its maintenance in TP901-1.

It was an unexpected result that the conservative exchange of Arg to Lys in amino acid 57 should result in defective lysogenization. Since the mutation is localised in a region which has been proposed to interact with the Mor protein [5,6], we hypothesized that the mutant CI protein might bind with higher affinity to the Mor protein, which should then be able to counteract repression by CI of the lytic promoter P_L_. Therefore this mutant in the *cI* gene was cloned without *mor* and it was shown that is was able to repress the P_L_ promoter almost as well as the wild type *cI* gene. This proves that the CI protein carrying the C8 mutation is functional. However, as the phenotype of the SD mutation in C18 can be explained by auto regulation of P_R_ we cannot exclude that auto regulation of P_R_ also is involved in the phenotype of C8. Further studies are thus needed to disclose whether C8 has an increased affinity for Mor. In conclusion our study shows that CI is the only protein involved in the lysogenization decision and the maintenance of the lysogenic state.

## Supporting Information

S1 Fig Physical map of clear plaque mutants. A physical map of ORF’s in the TP901-1 genome is shown below a size indicator. Localization of the mutations in TP 901-BC1034 is indicated by a red bar. The dark blue boxes indicate regions of similarity between TP901-1 and the prophage in 3101, the red box indicate the region that was recombined into TP 901-BC1034 and was verified by PCR. ERM indicates the position where the erythromycin resistance gene was inserted in TP 901-BC1034.
(TIF)

S1 Table List of additional mutations outside the lysogeny module of TP901-BC1034 in four of the clear plaque mutants. FS- Frame shift.
(DOCX)
