# Supplementary figures and images for: Clear Plaque Mutants of Lactococcal Phage TP901-1
Source: PLoS One. 2016 Jun 3;11(6):e0155233. doi: 10.1371/journal.pone.0155233 (PMC4892519; doi:10.1371/journal.pone.0155233)

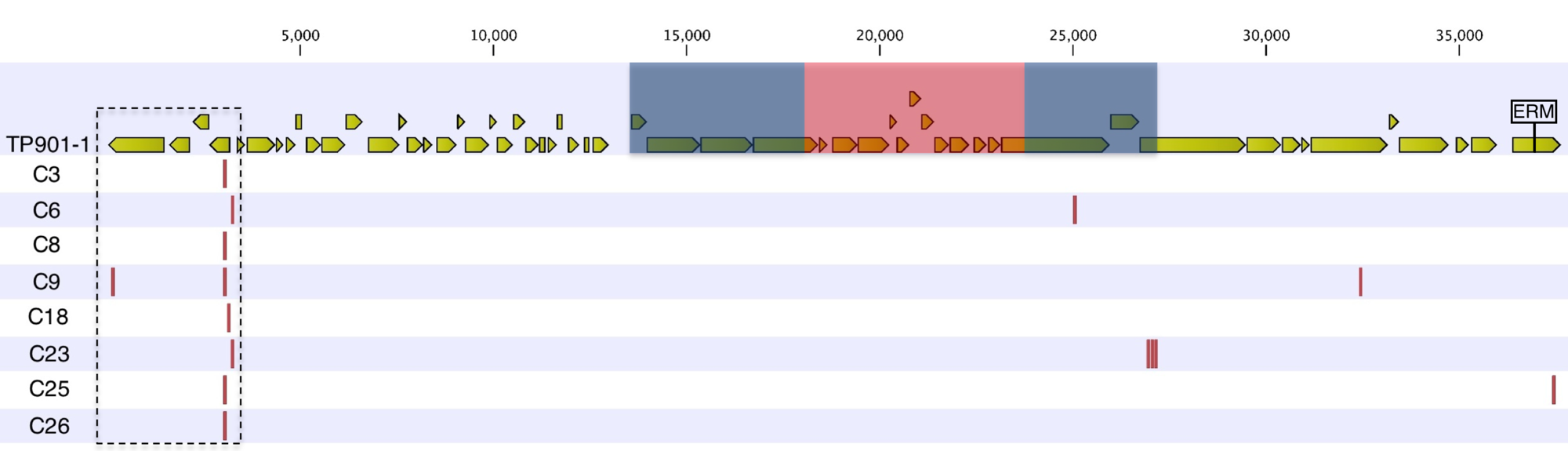

Supplement: S1 Fig — A physical map of ORF’s in the TP901-1 genome is shown below a size indicator. Localization of the mutations in TP 901-BC1034 is indicated by a red bar. The dark blue boxes indicate regions of similarity between TP901-1 and the prophage in 3101, the red box indicate the region that was recombined into TP 901-BC1034 and was verified by PCR. ERM indicates the position where the erythromycin resistance gene was inserted in TP 901-BC1034. (TIF) [file pone.0155233.s001.tif]
