# Supplementary material for: Clear Plaque Mutants of Lactococcal Phage TP901-1
Source: PLoS One. 2016 Jun 3;11(6):e0155233. doi: 10.1371/journal.pone.0155233 (PMC4892519; doi:10.1371/journal.pone.0155233)
Supplement: S1 Table — FS- Frame shift. (DOCX) [file pone.0155233.s002.docx]

**S1 Table.** **List of additional mutations outside the lysogeny module of TP901-BC1034 in four of the clear plaque mutants.** FS- Frame shift.

| Mutant | Mutation | Position in TP901-1 | Gene, function | Consequence |
| --- | --- | --- | --- | --- |
| C6 | G>A | 25001 | ORF45, *tmp* | Asp622Asn |
| C9 | A>- | 32443 | ORF51, *nps* | Asn433FS |
| C23 | TC>CA | 27069-70 | ORF47, *tal* | Gly117Lys |
| C23 | C>A | 27080 | ORF47, *tal* | Ser120Tyr |
| C23 | G>A | 27089 | ORF47, *tal* | Gly123Asp |
| C25 | T>C | 37406 | ORF56 | Silent |
